# A New Resistance Gene against Potato Late Blight Originating from *Solanum pinnatisectum* Located on Potato Chromosome 7

**DOI:** 10.3389/fpls.2017.01729

**Published:** 2017-10-04

**Authors:** Le Yang, Dongdong Wang, Yong Xu, Hua Zhao, Lei Wang, Xiaoli Cao, Yue Chen, Qin Chen

**Affiliations:** State Key Laboratory of Crop Stress Biology for Arid Areas, College of Agronomy, Northwest A&F University, Yangling, China

**Keywords:** potato, late blight, resistance gene, genetic map, collinearity analysis

## Abstract

Late blight, caused by the pathogen *Phytophthora infestans*, is one of the most devastating diseases of potato. Here, we describe a new single dominant resistance gene, *Rpi2*, from the Mexican diploid wild species *Solanum pinnatisectum* that confers high level and broad spectrum resistance to late blight. The *Rpi2* locus confers full resistance to complex isolates of *P. infestans*, for which race specificity has not yet been demonstrated. This new gene, flanked by the RFLP-derived marker *SpT1756* and AFLP-derived marker *SpAFLP2* with a minimal genetic distance of 0.8 cM, was mapped to potato chromosome 7. Using the genomic sequence data of potato, we estimated that the physical distance of the nearest marker to the resistance gene was about 27 kb. The map location and other evidence indicated that this resistance locus was different from the previously reported resistance locus *Rpi1* on the same chromosome from *S. pinnatisectum*. The presence of other reported resistance genes in the target region, such as *Gro1-4, I-3*, and three NBS-LLR like genes, on a homologous tomato genome segment indicates the *Rpi2*-related region is a hotspot for resistance genes. Comparative sequence analysis showed that the order of nine markers mapped to the *Rpi2* genetic map was highly conserved on tomato chromosome 7; however, some rearrangements were observed in the potato genome sequence. Additional markers and potential resistance genes will promote accurate location of the site of *Rpi2* and help breeders to introduce this resistance gene into different cultivars by marker-aided selection.

## Introduction

Late blight caused by *Phytophthora infestans* is one of the most devastating diseases of potato worldwide. Under favorable conditions for the pathogen, complete defoliation of a potato plant can occur in just a few weeks. The development and deployment of cultivars with genetic resistance is the most economical and eco-friendly approach for reducing yield losses due to late blight. Wild potato species are a valuable genetic pool for finding late blight resistant genes. The first paradigm came from the hexaploid Mexican wild species *Solanum demissum*. Eleven resistance (R) genes, named *R1* to *R11*, were identified in this wild species and introduced into *S. tuberosum*(Black, 1951; Black et al., 1953; Malcolmson and Black, 1966). However, these R genes conferred race-specific resistance and those that were introgressed into potato varieties were quickly overcome by the pathogen because of its high genetic variability (Wastie, 1991). Hence, new sources of resistance are required, especially those conferring race non-specific resistance to late blight.

The co-evolution of the pathogen and wild species in Central America indicated the possibility of finding resistance in species from Mexico such as *S. bulbocastanum, S. pinnatisectum*, and *S. trifidum*. A set of late blight resistance genes has already been identified in these species. Notably, in *S. bulbocastanum*, four different loci with broad spectrum late blight resistance have been identified, namely *Rpi-Blb1*/*RB* (Helgeson et al., 1998), *Rpi-blb2* (van der Vossen et al., 2005), *Rpi-blb3* (Park et al., 2005a), and *Rpi-apbt* (Park et al., 2005b). Recently, several other wild *Solanum* species have been reported as potential sources of resistance, such as *S. mochiquense* (Jones et al., 2013), *S. chacoense* (Vossen et al., 2011), and *S.* × *edinense* (De Vetten et al., 2014). Sustainable breeding efforts using these resistance sources have resulted in several new potato cultivars (Jo et al., 2014; Haesaert et al., 2015).

Extensive investigations have shown that the molecular basis of R gene resistance is a gene family characterized by two domains, the nucleotide binding site (NBS) and leucine-rich repeat (LRR) domains (Martin et al., 2003). The conserved nature of the motifs within these domains has been exploited to search for new resistance gene-like sequences or resistance gene analogs (RGAs) using a homology-dependent PCR-based approach (Kanazin et al., 1996; Leister et al., 1996; Chen et al., 1998; Hayes and Maroof, 2000). Many RGAs have been mapped to genomic positions containing known resistance specificities, and RGAs have been shown to be derived from known resistance genes (Collins et al., 1999). Thus, RGAs represent candidates for functional resistance genes. NBS-LRR genes can generally be divided into two distinct groups: one encoding an N-terminal domain with Toll/Interleukin-1 Receptor homology (TIR-NBS-LRR) and the other with an N-terminal coiled-coil motif (CC-NBS-LRR) (Martin et al., 2003). So far, over 20 late blight resistance genes, such as *R1, R2, R3a, R3b, RB, Rpi-blb2, Rpi-blb3, Rpi-abpt, Rpi-sto1, Rpi-pta1, Rpi-vnt1.1*, and *Rpi-vnt1.3*, which all belong to the CC-NBS-LRR class, have been cloned (Ballvora et al., 2002; Song et al., 2003; van der Vossen et al., 2003; Huang et al., 2005; Vleeshouwers et al., 2008; Foster et al., 2009; Lokossou et al., 2009; Pel et al., 2009). The publishing of the potato genome sequence derived from the *S. tuberosum* Group Phureja clone DM1-3 516 R44 (DM) accelerated the identification of 438 NB-LRR type genes from ∼39,000 potato gene models, and will increase the velocity of functional NB-LRR gene cloning from *Solanum* species (Jupe et al., 2012).

High level and broad spectrum late blight resistance has also been observed in the Mexican diploid wild species *S. pinnatisectum* (Menke et al., 1996; Chen et al., 2003). Compared with *S. bulbocastanum, S. pinnatisectum* has received less attention in late blight research. Kuhl et al. (2001) screened 13 accessions of *S. pinnatisectum* and found that most were resistant to late blight. Chen et al. (2003) revaluated the late blight resistance of *S. pinnatisectum* (PI275233) and found that it showed broad-spectrum resistance against various known *P. infestans* strains including the R9 isolate. They also found different levels of resistance among different accessions of *S. pinnatisectum*, suggesting the presence of different resistance genes. To date, only Kuhl et al. (2001) have reported the genetic analysis and identification of a single dominant resistance locus in *S. pinnatisectum* (PI253124), *Rpi1*, which was mapped to chromosome 7 in an interval of 14.6 cM between two RFLP markers, *CP56* and *TG20A*.

The hybridization barrier between *S. pinnatisectum* and cultivated potatoes had been overcome by the combination of the *Sli* gene and chromosome-doubling techniques (Sanetomo et al., 2014). Therefore, the wild species *S. pinnnatisectum* should receive more attention as a resource for potato late blight resistance breeding. The objective of this study was to characterize and map a late blight resistance gene from *S. pinnatisectum* (PI275233) through genetic linkage analysis and collinearity analysis. This gene may be useful for developing potato cultivars with broad spectrum resistance.

## Materials and Methods

### Plant Materials

A backcross population was developed by crossing the susceptible diploid *S. cardiophyllum* (PI186548) as the male parent with the resistant diploid *S. pinnatisectum* (PI275233). Several clones of a single resistant F1 individual, propagated through *in vitro* culture, were then backcrossed with the susceptible parent to generate a backcross mapping population.

The F_1_ and BC_1_ populations were maintained vegetatively from tubers following their first propagation from true seed. A total of 931 clones from the backcross population were selected to analyze the genetics of resistance to late blight using detached leaf methods at the first clonal generation (Chen et al., 2003).

### Detached Leaf Assay for Evaluating Late Blight Resistance

An inoculum was prepared from the P1801C.16 strain of *P. infestans* (US-8/A2 mating type) and diluted to a final concentration of 30,000 sporangia per ml. Inoculation and the detached leaf assay were performed according to Chen et al. (2003). Three compound leaves were excised for the late blight test, including the third to fifth leaves from the top on each plant’s main branch. Each compound leaf with five leaflets was inserted into prepared moist vermiculite in a plastic tray. The inoculum was sprayed onto the surface of all leaflets. Trays with inoculated compound leaves were incubated in a growth cabinet under an 18/6 h and 20/18°C day/night regime for about 15 days. The susceptible parent was inoculated as a susceptible check. Plant resistance was evaluated after 8 and 15 days. Disease severity was estimated using mean disease severity values (DSVs) of three compound leaves based on the percentage of leaf area with symptoms of late blight. Severity values were scored using a scale of 0–5 where 0 = no disease to <3%; 1 = 3–24%; 2 = 25–49%; 3 = 50–74%; 4 = 75–94%; and 5 = 95–100% infection. Plants with a DSV of 0 were classified as resistant and those with DSVs of 2–5 were classified as susceptible.

### AFLP Analysis

DNA was extracted from 100 mg of young leaves for each potato plant using a Genomic DNA Purification Kit (Promega, Fitchburg, WI, United States). Bulked segregant analysis (BSA) was used to screen for molecular markers associated with late blight resistance (Michelmore et al., 1991). Two susceptible bulks were constructed from 10 highly susceptible individuals and a resistant bulk was developed with equal amounts of DNA from 10 highly resistant individuals among the BC_1_ population. DNA markers were screened for the two susceptible bulks, the resistant bulk and the resistant parent plant.

AFLP analysis was performed as described by Vos et al. (1995) using *Eco*RI and *Mse*I as rare- and frequent-cutter enzymes, respectively. Genomic DNA digestion and ligation were conducted using an AFLP Core Reagent Kit (Invitrogen, Carlsbad, CA, United States) according to the instructions. A pre-amplification was carried out with 1-bp extension primer combinations (*Eco*RI+A/*Mse*I+C and *Eco*RI+A/*Mse*I+A) and the PCR products were diluted at a ratio of 1:30 with TE buffer. Selective amplification using primer combinations of *Eco*RI+3 and *Mse*I+3 was conducted and the products were separated on a 6% PAGE sequencing gel run at 100 W for 2.5 h after pre-electrophoresis for 30 min. The gel was stained by the silver-staining method (Bassam et al., 1991).

### DNA Sequencing and Analysis

AFLP fragments were excised from the dried silver-stained polyacrylamide gel and placed into microfuge tubes containing 30 μl distilled water. The samples were boiled for 10 min and centrifuged, and then 3 μl of the supernatant was used for PCR under the same conditions as those used for AFLP analysis. The PCR products were then inserted into the pGEM-T easy vector (Promega, Fitchburg, WI, United States) and sequenced. A search for sequences homologous to the AFLP fragments was conducted using the GenBank website^1^, and Clustal W2^2^ was used to compare the sequence homology.

### RGA Markers

The digestion of genome DNA were performed by two restriction enzymes, *Eco*RI and *Mse*I, according to the instructions of AFLP Core Reagent Kit. The primer combination *Eco*RI/*Mse*I was used to generate pre-amplification products. Then, the second amplification step was carried out with five primer combinations. The primer combinations were respectively combined by five P-loop based RGAs primers, S1 (5′-GGTGGGGTTGGGAAGACAACG-3′), S2 (5′-GGIGGIGTIGGIAAIACIAC-3′), AS1 (5′-CAACGCTAGTGGCAATCC-3′), AS2 (5′-IAAIGCIAGIGGIAAICC-3′) and AS3 (5′-IAGIGCIAGIGGIAGICC-3′) (Leister et al., 1996), with the *Eco*RI AFLP primer. PCR conditions were somewhat different from the standard AFLP procedure; 30 s DNA denaturation at 94°C, 30 s primer annealing at 55°C and a 1 min elongation step at 72°C (35 cycles). Prior to the cycling, the template DNA was denatured for 1 min at 94°C and the PCR was finalized by applying an extra 5 min elongation step at 72°C. The procedures for running the gel and fragment extraction were the same as described for AFLP section.

### Locus-Specific Marker Development

Locus-specific markers on chromosome 7 of potato and tomato^3^ were selected to develop PCR-based markers. Generally, the RFLP probe sequences were used as queries to search ESTs using the BLASTn program (Altschul et al., 1997). Then, the ESTs and RFLP probes were assembled with a criterion of more than 95% identity over a stretch of 40 nucleotides using SeqMan II (LASERGENE, DNASTAR, Madison, WI, United States). Primers were designed according to the assembled sequences guided by intron finder^4^ to amplify regions spanning introns and avoid placing primers in exon–intron boundaries. The PCR products were separated after digestion with one of the 4-bp cutter restriction enzymes *Taq*I, *Tru*l1, *Msp*1, *Rsa*1, and *Tai*l1.

The PCR amplification reactions were conducted in 20 μl reaction mixtures containing 10 mM TRIS-HCl, pH 8.3, 50 mM KCl, 2 mM MgCl_2_, 100 μM of each dNTP, 200 nM primers, approximately 20 ng template DNA and 1 Unit *Taq* DNA polymerase. The cycling program consisted of an initial 3 min denaturation step at 94°C, followed by 35 cycles of 94°C (30 s), 55°C (30 s), and 72°C (50 s), and a final 5 min extension step at 72°C. The PCR products were size-separated on a 2% agarose gel, stained with ethidium bromide, and visualized on a Gel Imaging system (Bio-Rad, San Diego, CA, United States).

### Linkage Analysis

Linkage analysis was performed using the software package MAPMAKER V3.0 (Lander et al., 1987). Markers and their corresponding distances (cM) were included in the framework map only if the LOD value for the ripple was >3. The Kosambi mapping function was employed to convert recombination frequencies to map distances in cM (Kosambi, 1943). Collinearity analysis results were visualized using Circos-0.67 (Krzywinski et al., 2009).

## Results

The BC_1_ generation produced 440 resistant plants and 491 susceptible plants. The segregation ratio fit a monogenic Mendelian inheritance model of 1:1 (resistant:susceptible) in the population (χ^2^ = 2.794, *P* = 0.095). This result suggested that a single dominant locus controlled the late blight resistance in *S. pinnatisectum* (PI275233). Subsequently, 164 susceptible and 101 resistant plants with extreme phenotypes were chosen for mapping.

### AFLP and AFLP-Derived Markers

In an attempt to find AFLP markers linked to the resistance locus, 324 *Eco*RI+3/*Mse*I+3 (196 E-A/M-C and 128 E-A/M-A) AFLP primer combinations were screened in the bulk material using a BSA strategy. Ten putative AFLP fragments were identified and segregation analysis in the BC_1_ population confirmed that seven of them were associated with the resistance locus. The two closest markers, *EAGCMCGA-450* and *EACAMAGG-330*, were determined to be linked to the resistance locus at distances of 1.2 and 0.8 cM, respectively.

**FIGURE 1:**
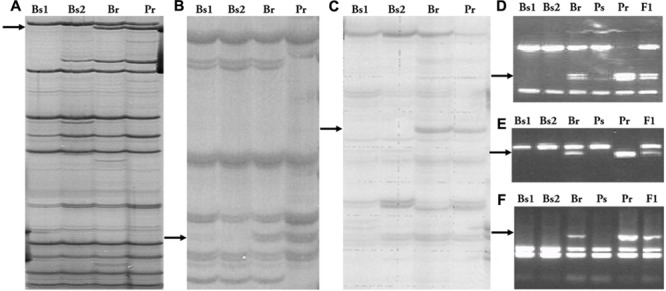
AFLP and RGA markers and their conversion into PCR-based markers. **(A)** Marker *EAGC/MCGA-450* and **(D)** its SCAR marker *SpAFLP1*. **(B)** Marker *EAAC/MATC-330* and **(E)** its SCAR marker *SpAFLP2*. **(C)** The *RGA1* marker and **(F)** its SCAR marker *SpGrol-1*. Bs1, Susceptible bulk 1; Bs2, susceptible bulk 2; Br, resistant bulk; Ps, susceptible parent; Pr, resistant parent.

These two AFLP fragments were cloned and sequenced. BLAST analysis showed that the sequence of *EAACMATC-330* had no similarity to any known sequence in GenBank, whereas *EAGCMCGA-450* hit four potato ESTs (BQ509088, BG600948, DV623421, DV623416). These four ESTs and two other potato ESTs were assembled into a 1720-bp contig with a complete coding region that showed high similarity to the Arabidopsis gene *GLUCAN SYNTHASE-like 7* (1e-139) in Blastx^5^) analysis. Based on this assembled sequence, a CAPS marker of *EAGCMCGA-450, SpAFLP1*, was developed (**Figures 1A,D**). In addition, *EAACMATC-330* was converted into the CAPS marker *SpAFLP2* (**Figures 1B,E**).

### Integration of *Rpi2* into the SGN Map

**FIGURE 2:**
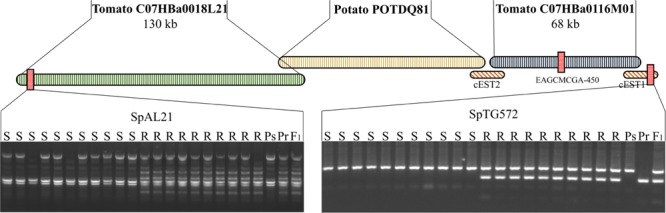
A contig built near the *Rpi2* locus. Three BACs (C07HBa0018L21, POTDQ81, and C07HBa0116M01) and two EST assembles (cEST1 and cEST2) overlapped and were assembled into a contig. Two markers located at either end of the contig were developed, *SpAL21* and *SpTG572*. Ps, Susceptible parent; Pr, resistant parent.

The 1720-bp contig of *EAGCMCGA-450* was used to search the high-throughput genomic sequence (HTGS) database with BLASTn^6^ and a tomato BAC (C07HBa0116M01) was identified (e-112). Annotation of this BAC (C07HBa0116M01) revealed a partial *VPS16*-like gene in the 3′ terminus. This partial gene sequence was used as a query to search the EST database with BLASTn and 23 matching ESTs were identified. All 23 ESTs were assembled to a 2.4-kb contig, named cEST1. BLAST analysis showed that this sequence had homology to an RFLP marker, *TG572* (e-120), which was mapped to tomato chromosome 7. Subsequently, a CAPS maker named *SpTG572* was developed according to this sequence and was shown to co-segregate with *SpAFLP1* (**Figure 2**).

The 5′ terminal sequence of BAC C07HBa0116M01 was used to build a 2-kb contig with 10 ESTs, named cEST2. This contig hit a potato BAC end sequence, POTDQ81TR. Using the other end sequence POTDQ81TF as a query, we identified a tomato BAC, C07HBa0018L21. A PCR marker, *SpAL21*, was developed based on the left end sequence of BAC C07HBa0018L21 and recombination was found between this marker and *SpTG572* (**Figure 2**).

**FIGURE 3:**
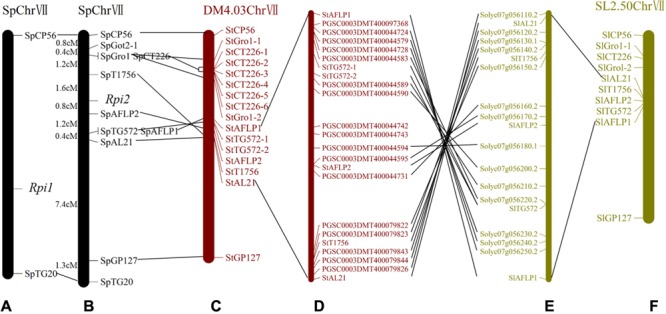
Genetic maps of the late blight resistance gene *Rpi2* and their correspondence to comparative genomic maps of DM and tomato. **(A)** A genetic map of *Rpi1* established by Kuhl et al. (2001). **(B)** A genetic map of *Rpi2* established in this study. **(C)** Homologous markers on *Solanum tuberosum* group Phureja DM1-3(DM) chromosome 7. **(D)** Annotated genes on DM chromosome 7 between *StAFLP1* and *StAL21*. **(E)** Annotated genes on *Solanum lycopersicum* (SL2.50) chromosome 7 between *SlAFLP1* and *SlAL21*. **(F)** Homologous markers on *S. lycopersicum* chromosome 7. Collinear loci are indicated by black lines.

*TG572* was near *I-3*, a gene for fusarium wilt resistance from the wild tomato species *Lycopersicon pennellii*, with a genetic distance less than 0.3 cM (Hemming et al., 2004). Two additional markers closely linked to *I-3, CT226*, and *Got2*, were converted to SCAR markers in our mapping population, and named *SpCT226* and *SpGot2*, respectively. Segregation analysis indicated *SpCT226* and *SpGot2* were proximal and distal with genetic distances of 2.8 and 3.2 cM, respectively (**Figure 3B**).

The flanking markers *TG572, T0810*, and *T1756* in the SGN map^7^ were developed into PCR-based markers and tested in the mapping population. The results delimited the resistance locus to the interval between *StAFLP2* and *SpT1756* on potato chromosome 7 (**Figure 3B**).

### An RGA Flanks the Resistance Locus

The RGA fingerprinting technique was used to identify functionally relevant markers linked to the resistance for late blight. An RGA fragment, RGA1, amplified by the AS2 primer (Leister et al., 1996) in combination with the E00 AFLP primer, did not exist in the two susceptible bulks but appeared in the resistant bulk and the resistant parent (**Figure 1C**). Sequence analysis of this 320-bp long fragment revealed homology to an RGA sequence previously mapped to the long arm of potato chromosome 7, *Gro1-5* (Leister et al., 1996). Therefore, this fragment was named *SpGrol-1*. A PCR marker was developed and segregation analysis indicated that *SpGrol-1* was 2.8 cM from the resistance locus (**Figure 1F**).

### Genetic Relationship between *Rpi1* and *Rpi2*

Previously, the late blight resistant locus *Rpi1*, also derived from *S. pinnatisectum*, was assigned to chromosome 7 flanked by two RFLP markers, *TG20* and *CP56* (Kuhl et al., 2001). To compare the map positions of *Rpi1* and our target resistance locus, the RFLP markers *TG20, CP56* and their interval marker *GP127*^8^ were converted into PCR-based markers. The marker information is listed in **Supplementary Table S1**. Segregation analysis of these converted PCR-based markers showed a link between the late blight resistance loci. The genetic distance between *CP56* and *TG20* was 15.1 cM, similar to the map of Kuhl et al. (2001) (14.6 cM). However, there was an obvious difference in the genetic distance between the markers linked to the resistance genes. *CP56* and *TG20* were 4.0 and 11.1 cM away from our target gene, respectively, which was different to the genetic distances of *Rpi1* to these two markers (9.4 and 5.2 cM, respectively) (**Figures 3A,B**). Therefore, our resistance gene was called *Rpi2*.

Although the two resistance genes were derived from the same species, they came from two different accessions; *Rpi1* from PI235214 and *Rpi2* from PI 275233. To further identify genetic differences between the accessions PI275233 and PI235214, we screened all of the markers and found that the two accessions had different genotypes at two loci, *SpAFLP2* and *SpCT226*.

### Collinearity Analysis of Target Chromosome Regions between Potato and Tomato

**FIGURE 4:**
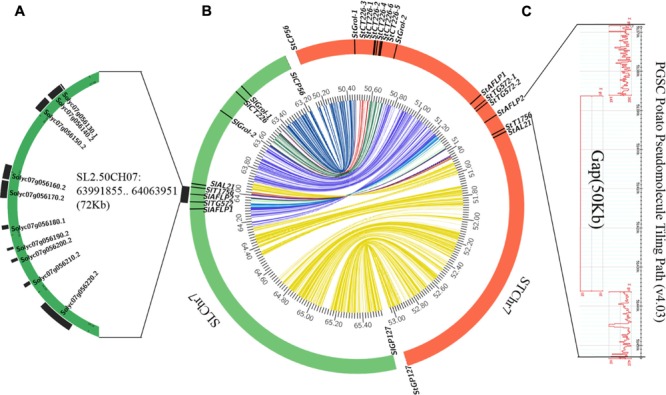
Collinearity analysis of target chromosome regions between potato and tomato. **(A)** The chromosome region between *SlT1756* and *SlTG572*, which contains 10 annotated genes in the tomato genome. **(B)** Collinearity analysis of *Rpi2*-related chromosome regions between potato (DM v4.03) and tomato (SL2.50). **(C)** A 50-kb gap between *StT1756* and *StAFLP2* in the potato genome.

The molecular marker sequences were used as queries to search for homologous loci in the genome sequence databases of potato and tomato. The majority of markers linked to *Rpi2* showed homology to STChr7 and SLChr7, and were included in two orthologous genomic regions spanning 2.9 and 2.4 Mb, respectively (**Figure 4B**). Specifically, 9 of the 11 molecular markers mapped in the *Rpi2* genetic linkage map generated hits to 16 homologous loci in STChr7 and 10 loci in SLChr7 (**Figures 3C,F**). Because cloning and sequencing failed, homologous loci of *SpGot2-1* and *SpTG20* were not found.

Comparative genomic analysis revealed that 14 of the 17 annotated genes in potato between *StAFLP1* and *StAL21* had similarity to corresponding regions in tomato (**Figures 3D,E**), again revealing high levels of collinearity in the *Rpi2* region between potato and tomato. Furthermore, the order of these similar genes was highly conserved but reversed. Segmental inversion, which was a reasonable explanation for the reverse order of the conserved genes, was observed on STChr7 between *StAFLP1* and *StAL21* compared with the *Rpi2* linkage map and SLChr7.

Scanning the Spud DB Genome Browser for Potato (*Solanum tuberosum* group Phureja DM1-3) PGSC v4.03 Pseudomolecules^9^ suggested the physical distance between *StAFLP2* and *StT1756* was about 84 kbp. Consequently, we estimated that the physical distance between *Rpi2* and *SpAFLP2* was about 28 kbp by referring to their genetic distance. Unfortunately, there was a 50-kbp gap in this potato genome region that potentially contained the homologous gene of *Rpi2* (**Figure 4C**). However, the homologous segment in the tomato genome was assembled completely, in which 3 of 10 loci **Table 1** were annotated as NBS-LRR class disease resistance proteins (Accession nos. Solyc07g056180.1, Solyc07g056190.2, and Solyc07g056200.2) (**Figure 4A**).

**Table 1:** Annotated genes in the tomato genome region between *SlT1756* and *SlTG572.*

Name	Location on SL2.50ch07	Description	InterPro domain
Solyc07g056130.1	63988594..63988983	Unknown protein	–
Solyc07g056140.2	63989209..63993821	Glucose-1-phosphate adenylyltransferase	IPR011831
Solyc07g056150.2	63994885..63999176	Ras-related protein Rab-2-A	IPR003579
Solyc07g056160.2	64018080..64022506	Cytochrome P450	–
Solyc07g056170.2	64023009..64028326	Subtilisin-like protease	IPR015500
Solyc07g056180.1	64036615..64037860	NBS-LRR class disease resistance protein	–
Solyc07g056190.2	64043548..64044315	NBS-LRR class disease resistance protein	–
Solyc07g056200.2	64047159..64048119	NBS-LRR class disease resistance protein	–
Solyc07g056210.2	64055352..64056480	Unknown protein	–
Solyc07g056220.2	64063658..64074684	Vacuolar sorting-associated protein	IPR016534

## Discussion

The short-lived R genes from *S. demissum* prompted potato breeders and geneticists to look for resistance genes in other wild *Solanum* species (Van Soest et al., 1984; Colon and Budding, 1988; Douches et al., 2001). High-level resistance has been found in several diploid Mexican species, including *S. bulbocastanum* and *S. pinnatisectum* (Helgeson et al., 1998; Kuhl et al., 2001; Chen et al., 2003). These species may have adapted to coexist with highly complex and dynamic *P. infestans* populations (Niederhauser, 1953; Niederhauser et al., 1954). Genetic mapping studies indicated that the resistance in both *S. bulbocastanum* and *S. pinnatisectum* might be conferred by a single gene or a few dominant genes (Naess et al., 2000; Kuhl et al., 2001). Here, we identified a single dominant late blight resistance gene from the wild potato species *S. pinnatisectum* (PI 275233) and mapped it to an interval of 2.4 cM on the long arm of chromosome 7.

### A Hotspot of Resistance Genes on Potato Chromosome 7

Accumulated evidence has suggested that resistance loci are not distributed randomly along chromosomes. Several hotspots for resistance genes have been described in *Solanum* species. For instance, at least five R genes against diverse pathogens have been mapped to the *GP21*–*GP179* interval on chromosome 5 in different genetic backgrounds; *Gpa* and *Grp1* conferring resistance to potato cyst nematodes (Kreike et al., 1994; van der Voort et al., 1998), *Nb* and *Rx2* conferring resistance to potato virus X (Ritter et al., 1991; DeJong et al., 1997), and *R1* conferring resistance to *P. infestans* (Leonards-Schippers et al., 1992). In the current study, we found that *Rpi2* was located in a major cluster on the long arm of chromosome 7 in which several R genes have been mapped, including *Rpi1* conferring resistance to *P. infestans, Gro1-4* conferring resistance to *Globodera rostochiensis*, and *I-3* conferring resistance to *Fusarium oxysporum* (Bournival et al., 1989; Ballvora et al., 1995; Kuhl et al., 2001; Paal et al., 2004; Ruggieri et al., 2014; Catanzariti et al., 2015). Clearly, this region is another hotspot for resistance genes, and can be expected to contain more resistance genes.

Resistance loci regions are usually enriched in NBS-LRR homologs. For instance, there are at least 13 TIR-NBS-LRR sequences clustered across more than 400 kb in the locus *Gro1* (Paal et al., 2004) and one of them, *Gro1-4*, has been shown to be responsible for a resistance trait. In this research, we used the RGA profiling strategy to identify an RGA fragment, *SpGrol-1*, linked to the resistance locus. Sequence analysis showed that *SpGrol-1* belonged to the TIR-NBS-LRR family, and that the most similar sequence was *Gro1-5*, a gene at the *Gro1* locus. However, mapping analysis showed this RGA was proximal to *Rpi2* with a genetic distance of 2.8 cM.

Resistance gene analogs are generally clustered in the genome (Meyers et al., 1998; Michelmore and Meyers, 1998). Clusters of R genes can be tightly organized or spaced over several megabases of sequence (Meyers et al., 1998; Noël et al., 1999). We thought that similar *Gro1*-like sequences might be present in our resistance locus. Hence, we designed a set of primers according to an alignment of 13 *Gro1* sequences and developed three PCR markers. However, all of these markers co-localized to *SpGrol-1* (data not shown). This indicated that there was more than one *Gro1*-like sequence and that the *Rpi2* gene might not be a *Gro1*-like gene.

A similar observation was also described for the resistance gene *I-3* from the wild tomato *L. pennellii* (Hemming et al., 2004). *I-3* co-segregated with RGA St332; however, RGA St332 was ruled out as a candidate gene for *I-3* because it was a single-copy pseudo gene in *L. pennellii*. *I-3* was flanked by two RFLP markers TG572 and CT226 in an interval of 0.6 cM. That *Rpi2* and *I-3* share the flanking markers CT226 and TG572 supports that these two genes are in a syntenic region.

### Comparative Sequence Analysis of the *Rpi2* Region

Comparative genomics between potato and tomato facilitated the mapping and isolation of the late-blight R genes *R3a* and *Rpi-blb2* from potato in a previous study, as these genes were mapped to regions of the potato genome that were syntenic to previously cloned gene loci (*I2* and *Mi*, respectively) in tomato (Huang et al., 2005; van der Vossen et al., 2005). Recently, both the potato and tomato genomes have been sequenced (Xu et al., 2011; Sato et al., 2012). This sequence information should greatly accelerate the cloning of the *Rpi2* gene through comparative genomics.

Comparing the homologous regions in the potato and tomato genomes, the genetic linkage map of *Rpi2* showed high uniformity except that a chromosome inversion had occurred in the sequenced DM genome (**Figure 3**). Although this inversion may be a result of chromosomal variation during evolution, incorrect sequencing or assembly could equally have led to the observed recombination because short reads, a large amount of repetitive sequence, the sequence GC composition and other effects can impede uniform and complete sequencing coverage along the genome (Maiti and Bouvagnet, 2001; van Hijum et al., 2005; Aird et al., 2011; Schatz et al., 2012; Berlin et al., 2015). The gap between *SpAFLP2* and *SpT1756* on StChr7 indicated the accuracy of the assembly in the *Rpi2*-related region was not sufficiently high. In other words, the fragment inversion observed by comparative analysis was not sufficient evidence to demonstrate chromosome inversion. Furthermore, the lack of sequence information between the flanking markers prevented us from obtaining candidate genes from the DM genome data. Therefore, constructing a higher quality genome assembly for the *Rpi2*-related region requires enhanced approaches.

At present, an effort to introgress disease resistance genes from *S. pinnatisectum* into potato is being carried out to develop resistant cultivars. Because of the ploidy level barrier and endosperm balance number incompatibility, it is difficult to transfer resistance traits from *S. pinnatisectum* to cultivated potato. Fortunately, the hybridization barrier between *S. pinnatisectum* and cultivated potatoes can be overcome by embryo rescue, protoplast fusion, and chromosome-doubling techniques (Chen et al., 2008; Sanetomo et al., 2014). Our molecular markers could help breeders to introduce this resistance gene into different cultivars by marker-assisted selection.

## Author Contributions

LY and DW: Conducted the experiments, analyzed the data, and wrote the manuscript. YX: Identified the resistant parental line and made the cross. LW and XC: Participated in detecting molecular makers and contributed to the genotyping experiment. HZ: Assisted in analyzing the data. YC and QC: Conceived and directed the project and revised the manuscript.

## Conflict of Interest Statement

The authors declare that the research was conducted in the absence of any commercial or financial relationships that could be construed as a potential conflict of interest.

## Footnotes

1 https://www.ncbi.nlm.nih.gov/genbank/

2 http://www.ebi.ac.uk/Tools/msa/clustalw2/

3 https://solgenomics.net/cview/index.pl

4 http://ftp.sgn.cornell.edu/tools/intron_detection/find_introns.pl

5 https://blast.ncbi.nlm.nih.gov/Blast.cgi?PROGRAM=blastx&PAGE_TYPE=BlastSearch&LINK_LOC=blasthome

6 https://blast.ncbi.nlm.nih.gov/Blast.cgi?PROGRAM=blastn&PAGE_TYPE=BlastSearch&LINK_LOC=blasthome

7 https://solgenomics.net/cview/index.pl

8 https://solgenomics.net/marker/SGN-M16437/details

9 http://solanaceae.plantbiology.msu.edu/cgi-bin/gbrowse/potato/

## Supplementary Material

TABLE S1 Information of molecular markers involved in this study.
